# Extraovarian Granulosa Cell Tumor of Mesentery: A Case Report

**DOI:** 10.4061/2010/292606

**Published:** 2010-03-04

**Authors:** Manjiri R. Naniwadekar, N. J. Patil

**Affiliations:** ^1^Department of Pathology, Krishna Institute of Medical Sciences University, Karad 415110, India; ^2^Naniwadekar Hospital, Market Yard, Shaniwar Peth, District Satara (Maharashtra), Karad 415110, India

## Abstract

Extraovarian granulosa cell tumor (GCT) is a very uncommon tumor, assumed to arise from the ectopic gonadal tissue along the embryonal route of the genital ridge. A 54 years old female patient presented with a mass and acute pain in abdomen. Exploratory laparatomy revealed hemoperitoneum with a large mesenteric mass measuring 13 × 12 cm in size, showing extensive areas of haemorrhages. Histopathological examination of the excised mass showed features of adult-type GCT. As the patient had a history of hysterectomy with bilateral salpingo-oophorectomy 10 years ago for ‘‘leiomyoma” with no evidence of GCT of the ovary in the histopathology report, a diagnosis of extraovarian GCT was made. A diagnosis of extraovarian GCT should be carried out after excluding any previous history of GCT of the ovary. Tumor rupture with haemoperitoneum is a well-known complication of GCT. Extraovarian GCT is a rare tumor with only 10 cases reported in literature. The case is presented for its rarity.

## 1. Introduction

Granulosa cell tumors (GCT) are the most common malignant sex cord—stromal tumors of the ovary [1]. They can arise in locations other than the ovary and may be derived from the mesenchyme of the genital ridge [2]. Women who have undergone oophorectomy may have the potential to develop GCT [2]. The primary extraovarian GCT is extremely rare tumor [3]. In the English literature only 10 cases have been reported to date [3, 4], including one case arising in a mullerian cyst of the broad ligament [5]. Ours is the eleventh case and third case from India [4, 6].

## 2. Case Report

A 54 years old female presented with mass and acute pain in abdomen. She gave a history of hysterectomy with bilateral salpingo-oophorectomy 10 years ago for uterine leiomyoma. Per abdominal examination revealed a vague mass palpable in epigastric and periumbilical region. On investigation, her hemoglobin was 8.6 gram% and total WBC count was 15,500/cumm with neutrophilia. RBC morphology was hypochromic and microcytic on peripheral smear. Sonography of the abdomen showed a 12 × 12 cm-sized solid heterogeneous mass in the mesentery. Exploratory laparatomy revealed hemoperitoneum with a large mesenteric mass measuring 13 × 12 cm in size showing extensive areas of haemorrhages. The mass was removed. 

**Figure 1:**
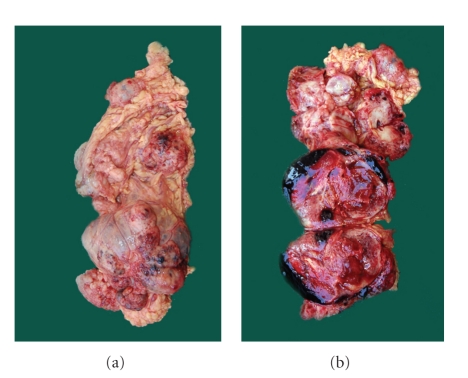
grayish brown, soft, and nodular mass with haemorrhagic areas measuring 13 × 11 cm. Cut section showed solid homogenous grayish white tumor with areas of haemorrhages.

 Gross findings—the mass was grayish brown, soft, and nodular with haemorrhagic areas measuring 13 × 11 cm. Cut section revealed solid homogenous grayish white tumor with small cystic areas and areas of haemorrhages (Figure 1). 

**Figure 2:**
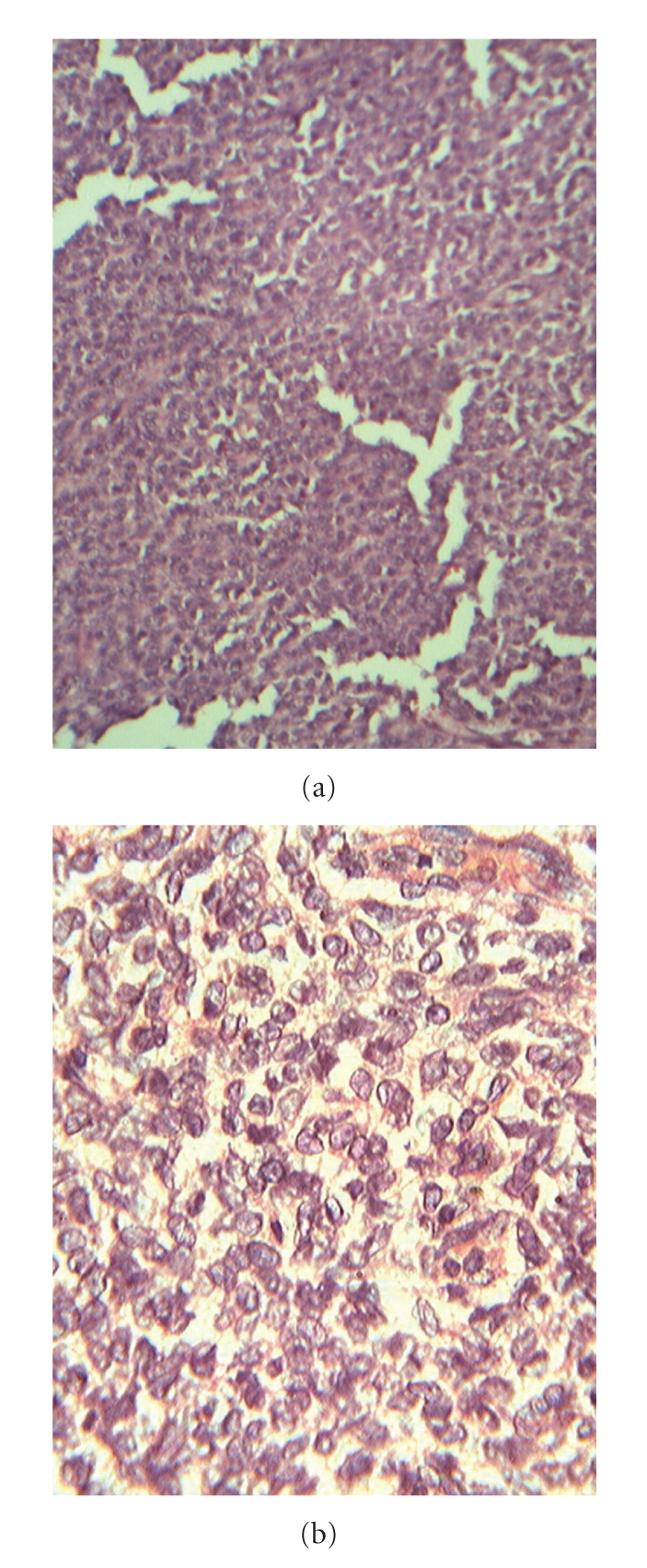
Microphotogaph showing a tumor composed of small round to oval neoplastic cells with predominantly diffuse patterns. The cells showed scanty cytoplasm and round to oval nuclei with nuclear grooves—coffee bean nuclei (H&E ×100-(a) and ×400-(b)).

 Microscopic findings—sections from the tumor showed small round to oval neoplastic cells with predominantly diffuse and trabecular patterns. The cells showed scanty cytoplasm and round to oval nuclei with nuclear grooves—coffee bean nuclei (Figure 2). With the typical histopathological features, a diagnosis of GCT (adult type) of mesentery was made. The patient having a history of hysterectomy for leiomyoma, histopathology reports were reviewed which showed no evidence of GCT of ovary. Immunohistochemistry (IHC) for inhibin and EMA was done. The tumor was positive for inhibin and negative for EMA, thus confirming the diagnosis of extraovarian GCT (adult type) of mesentery.

## 3. Discussion

Granulosa cell tumors (GCT) are the malignant sex cord—stromal tumors of the ovary. They constitute 1-2% of all ovarian tumors [1]. They can recur or metastatsize many years after initial treatment. Rarely GCT can develop at an extraovarian site. Possibility of metastasis has to be ruled out before diagnosis of extraovarian GCT. Extraovarian granulosa cell tumor is a rare tumor with only ten cases reported in English literature [4]. Two cases were reported from India [4, 6]. Extraovarian granulosa cell tumor can develop in retroperitoneum [4, 7], broad ligament [5], mesentery, omentum, liver, adrenals, and so forth, [4]. Histogenetic origin is thought to be from ectopic gonadal stromal tissue from the mesonephros [4]. 

 GCTs vary in their gross appearance. Most are partly cystic and partly solid. Intracystic hemorrhage is common [1]. Microscopically, the tumor cells resemble normal granulosa cells. They are small with uniform round or oval hyperchromaic nuclei with finely granular chromatin and longitudinal nuclear grooves or folds [1]. They show microfollicular, macrofollicular, trabecular or diffuse pattern [1]. Similar histologic findings were observed in the present case, with a predominantly diffuse and trabecular pattern. 

 Extraovarian GCT should be differentiated from other metastatic carcinomas of ovary having similar morphology. Inhibin and EMA can help in differentiating these tumors. GCT is positive for inhibin and negative for EMA. GCT also has to be differentiated from other tumors such as small cell carcinoma, undifferentiated carcinoma, endometrial stromal sarcoma, carcinoid, and lymphoma [1]. These tumors do not show positivity for inhibin. IHC for CK, EMA, LCA, CD99 and Chromogranin can help in diagnosing and differentiating these tumors. GCT does not show positivity for EMA, LCA, and Chromogranin. 

 In the present case, the patient had a history of hysterectomy, with bilateral salpingo-oophorectomy 10 years ago for “leiomyoma” with no evidence of GCT of the ovary in the previous histopathology report. The patient presented with mass in abdomen and pain due to tumor rupture with haemoperitoneum, which is a well-known complication of GCT. Hormonal studies were not done, as the diagnosis of GCT was not suspected. The histopathology features of the present tumor are typical of GCT, viz-small pale, round to oval granulosa cells with diffuse histological pattern and characteristic “coffee-bean” nuclei. The tumor showed positivity for inhibin while EMA was negative, thus confirming the diagnosis of GCT. 

 The case is reported for its rarity and to describe its relevance to the histologic origin and in clinical practice. Women with H/O oophorectomy may develop extraovarian GCT. Diagnosis is made by characteristic histologic features and by excluding previous H/O GCT of ovary. Immunostains like inhibin help in definitive diagnosis.
